# Automated Competitive Protein‐Binding Assay for Total 25‐OH Vitamin D, Multicenter Evaluation and Practical Performance

**DOI:** 10.1002/jcla.21793

**Published:** 2014-08-17

**Authors:** Jos PM Wielders, Graeme F. Carter, Heike Eberl, Gary Morris, Heinz Jürgen Roth, Christian Vogl

**Affiliations:** ^1^ Meander Medisch Centrum Amersfoort The Netherlands; ^2^ Healthscope Pathology Clayton Victoria Australia; ^3^ Roche Diagnostics GmbH Penzberg Germany; ^4^ Southern IML Pathology Wollongong New South Wales Australia; ^5^ Limbach Laboratories Heidelberg Germany

**Keywords:** electrochemiluminescence, Elecsys vitamin D total, vitamin D binding protein, VDBP, vitamin D, 25‐hydroxyvitamin D, robustness

## Abstract

**Background:**

The Roche Elecsys Vitamin D Total competitive protein‐binding assay uses recombinant vitamin D binding protein for measuring 25‐hydroxyvitamin D (25‐OHD), which is different from commonly used antibody assays.

**Methods:**

The assay, standardized against LC‐MS/MS, was tested at four sites. Evaluation included precision; between‐laboratory variability; functional sensitivity; correlation to LC‐MS/MS, HPLC, and immunoassays; as well as robustness, traceability, and EQAS performance.

**Results:**

Precision testing showed within‐run coefficient of variations (CVs) of ≤7%, within‐laboratory CVs of <9.5%, between‐laboratory precision CVs of ≤10.1%, and a functional sensitivity below 9.8 nmol/l (at CV 12.9%). The assay showed equivalent 25‐OHD levels for matched serum and plasma samples, good reagent lot‐to‐lot consistency in pooled sera over time, and good agreement with HPLC (relative bias −8.8%). Comparison with LC‐MS/MS methods yielded relative biases of −15.4, −13.5, −10.2, and 3.2%. Comparison against immunoassays showed a relative bias of 14.5% (DiaSorin Liaison) and −58.2% (IDS‐iSYS). The overall mean results in 2 years DEQAS was 102% of the ALTM. In a certified reference patient panel, the average bias was <4% for the sum of 25‐OHD2 and 25‐OHD3.

**Conclusion:**

The Elecsys Vitamin D Total assay demonstrated good overall performance and is, according to present standards, very suitable for automated measurement of 25‐OHD.

## INTRODUCTION

Measurement of 25‐hydroxyvitamin D (25‐OHD) is increasingly used to assess and monitor vitamin D status and guide supplementation to preserve bone health and to prevent extraskeletal hypovitaminosis D effects such as fatigue and muscle complaints. An association of vitamin D status with cardiovascular diseases, cancer, and autoimmunity has been described 1, 2, 3, 4, 5, 6.

The two most important forms of vitamin D are vitamin D3 (cholecalciferol, naturally occurring in humans and animals) and vitamin D2 (ergocalciferol, naturally occurring in plants and present in supplements in some countries). Both forms follow the same metabolic pathway in the human body. The first step is hydroxylation in the liver, resulting in the stable 25‐OHD forms (25‐OHD3 and 25‐OHD2). These forms circulate through the body bound to the vitamin D binding protein (VDBP) and are subsequently converted to the biologically active metabolite 1,25‐dihydroxyvitamin D in the kidneys as well as locally in many tissues and cells 1, 6, 7.

It is widely accepted that the 25‐OHD concentration in plasma/serum is the best indicator of vitamin D status, measured as the sum of 25‐OHD3 and 25‐OHD2. Most of the total 25‐OHD in plasma or serum is represented by 25‐OHD3, whereas 25‐OHD2 is present in significant amounts only in subjects taking vitamin D2 supplements 3, 8, 9.

Currently, most experts agree that vitamin D deficiency should be defined as 25‐OHD concentrations <20 ng/ml (<50 nmol/l) 10, and sufficiency as 20 ng/ml (50 nmol/l) 11 or greater. Even 30 ng/ml (75 nmol/l) is recommended by some clinical societies 12, 13 as desirable.

Commonly used 25‐OHD‐measurement methods are competitive immuno‐ or protein‐binding assays, either fully automated or manual assays, or chromatographic techniques such as high‐performance liquid chromatography (HPLC/UV) and LC coupled with tandem mass spectrometry (LC‐MS/MS). LC‐MS/MS is considered as the reference method for 25‐OHD determination; however, its use in routine testing is limited due to the high level of expertise required and the time‐ and labor‐intensive nature of the method. Conversely, automated immuno‐ or protein‐binding assays are rather simple methods, allowing high throughput and consolidation with immunochemistry testing on routine bulk analyzers.

Procedures for 25‐OHD detection differ in features such as sample‐preparation steps for complete and consistent release of 25‐OHD from VDBP, separation of the analyte from other components in the matrix (immunoassay or LC), detection system (light absorption or emission, radioactivity, or mass detection), and signal‐generating system (chemiluminescence or enzymatic reaction, radioactive labeling). These differences cause method‐specific effects and trouble comparison results between laboratories 2, 5, 7, 14, 15. Moreover, reports on the high variability and different standardization of assays raised questions about assay reliability and possible impact on medical decisions 16, 17, 18, 19. Recently introduced standards SRM 972 (vitamin D in human serum) and SRM 2972 (ethanolic solutions of either 25‐OHD2 or 25‐OHD3) from the National Institute of Standards and Technology (NIST) for use in quality control or as primary assay calibrator 5, 14, 17, 18, 19 should improve reliability and traceability. This article describes the evaluation of an automated competitive protein‐binding assay in a multicenter study with special focus on traceability and performance in routine laboratory testing.

## MATERIALS AND METHODS

### Development and Principles of the Elecsys Vitamin D Total Assay

The Elecsys Vitamin D Total assay is a competitive protein‐binding assay using a recombinant VDBP (recVDBP) that captures both 25‐OHD3 and 25‐OHD2, allowing the quantitative determination of total 25‐OHD. The recVDBP is based on the human amino acid sequence and is expressed in suspension‐adapted human embryonic kidney cells (HEK293 cell line) propagated in a chemically defined medium. Signal generation is based on the electro‐chemiluminescence technology, generating high sensitivity 20. The method principle is illustrated in Supplemental Figure 1. The required sample volume is 15 μl and the overall duration of the assay is 27 min.

**Figure 1:**
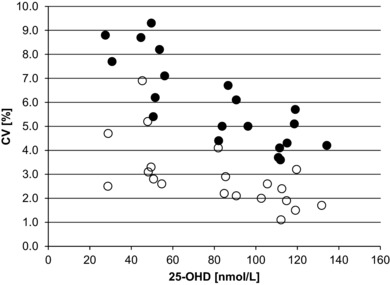
Within‐run and within‐laboratory (intermediate) precision. Within‐run precision (open circles) of the Elecsys Vitamin D Total assay was determined by measuring in a single run 21 aliquots of each of two assay controls (PreciControl Varia (PCV1 and PCV2)) and three serum pools (SP1, SP2, SP3, prepared separately by each of the five study sites). Within‐laboratory (intermediate) precision (filled circles) was evaluated for a total of 84 aliquots of each of the two control standards (PreciControl Varia (PCV1 and PCV2)) and three serum pools (SP1, SP2, SP3).

The Elecsys assay is standardized against LC‐MS/MS calibrated using NIST standard reference material SRM 2972 21. The LC‐MS/MS used for standardization of the Elecsys assay was based on the method described by Vogeser et al. 2004 22 and modified to introduce the following: (i) co‐detection of 25‐OHD3 as well as 25‐OHD2; (ii) higher labeled 25‐OHD3 (D6–25‐OHD3) as internal standard; (iii) longer washing steps of the trap and analytical columns to decrease matrix effects; (iv) two multiple‐reaction monitoring transitions with optimized collision energies for each analyte to increase specificity of the method; (v) six‐point external calibration, ranging from ∼1.5 nmol/l to 298.3 nmol/l for 25‐OHD2 and 2.1 to 418.5 nmol/l for 25‐OHD3, respectively 23; and (vi) NIST SRM 2972 as direct calibrators and NIST SRM 972 as quality‐control material. The SI units for vitamin D concentration are converted to traditional units by the equation 25‐OHD nmol/l = 25‐OHD ng/ml × 2.5.

### Participating Laboratories and Study Design

The multicenter evaluation was performed at four laboratories in Europe and Australia (Table 1). In addition, LC‐MS/MS measurements on samples from the sites in Australia (Clayton, Wollongong) and Munich were performed at a medical laboratory in Heidelberg, Germany. As this methods allows separation of the 3‐epimer of 25‐OHD3, its concentration was added to the 25‐OHD3 and 25‐OHD2 concentrations to ensure comparability to the method described above. The median concentrations for the three sample sets were 3.25 (2.25–17.8), 2.88 (1.75–11.3), and 2.5 (2.25–2.75) nmol/l. All participating laboratories have substantial experience in performing vitamin D testing. The performance of the Elecsys assay was assessed on Modular Analytics E170, cobas e 601, and cobas e 411 analyzers (Roche Diagnostics, Germany; Table 1).

**Table 1:** Participating Laboratories and Detection Methods

Participating laboratory (sample number)	Roche system	Experiments performed	Comparison method(s)
Clayton, Australia (*N* = 199)	cobas e 601	Precision Lot‐to‐lot comparison	LC‐MS/MS^a^
Wollongong, Australia (*N* = 166)	Modular analytics E170	Precision Lot‐to‐lot comparison	LC‐MS/MS^a^ Liaison DiaSorin
Munich, Germany (*N* = 164)	cobas e 411	Precision	LC‐MS/MS^a^ IDS‐iSYS
Amersfoort, The Netherlands (*N* = 200)	cobas e 601	Precision Lot‐to‐lot comparison, Serum/plasma comp functional sensitivity	LC‐MS/MS^b^ HPLC

a LC‐MS/MS testing at the laboratory in Heidelberg, Germany, with a modified method (25): Waters Xevo TQ‐S using calibrators from Chromsystems and NIST SRM 972.

b LC‐MS/MS testing at Roche Diagnostics, Penzberg, Germany, with a modified method (22): Thermo Scientific TSQ Quantum Ultra EMR using NIST SRM 2972 for calibration.

At all participating laboratories, within‐run precision, intermediate precision (according to CLSI EP5‐A2 24, and between‐laboratory variability and method comparison to other 25‐OHD methods were tested. Selected laboratories assessed functional sensitivity, lot‐to‐lot consistency, and serum/plasma correlation (Table 1).

### Patient Samples

Sample material used was from residual samples; all samples were anonymized and divided into 300 μl aliquots. A minimum of three aliquots was prepared, frozen as soon as possible, and stored at −20/−80°C. For sample pools, the final analyte concentration of the pool was verified on the Elecsys/cobas e instruments with the evaluation reagent. The whole pool was centrifuged and stored either in appropriately sized subpools, or directly in Hitachi sample cups at −20/−80°C. Samples were thawed using a water bath set at room temperature and homogenized by gently inverting the tubes. The samples were then checked for clots. Samples prepared for a run were kept at 4°C before being placed on the sample rotor. Because samples were obtained from patients in countries that do not use vitamin D2 supplements, the 25‐OHD2 concentration is negligible.

### Precision

Within‐run and within‐laboratory (intermediate) precision were assessed with two assay controls (Roche Diagnostics, Germany), and with three native serum pools (SP1, SP2, SP3) generated by each participating site. The levels of the PreciControl Varia controls were ∼50 nmol/l (PCV 1) and ∼100 nmol/ (PCV 2). The levels of 25‐OHD in the serum pools were in the range 28 to 50 nmol/l (SP1), 82 to 91 nmol/l (SP2), and 96 to 134 nmol/l (SP3). Within‐run precision was analyzed using 21 aliquots of each of the controls and sample pools in one run.

Within‐laboratory (intermediate) precision was performed using four aliquots per level of each of the controls and sample pools, which were randomized and analyzed in two runs per day for either 21 or 10 days. At the two study sites (Clayton and Munich), samples were randomized for two assay runs per day for 21 days, measuring two aliquots of each of the five samples per run. Two other study sites (Wollongong and Amersfoort) performed a shortened 10‐day precision experiment (*N* = 40). To simulate routine laboratory conditions, the samples were randomized in each run. Each run also contained 30 serum “dummy” samples that were not taken into account for the precision analysis.

Between‐laboratory precision was assessed using three serum pools (SP4, SP5, and SP6) provided by Roche Diagnostics with concentrations of 37.5, 50, and 100 nmol/l, respectively. Two aliquots of each of the serum pools SP4, SP5, and SP6 were tested in ten separate runs, with a maximum of two runs per day. The recovery of 25‐OHD for the three pools was calculated by comparing the individual median concentration determined at each study site with the respective median concentration of all sites.

### Functional Sensitivity

Functional sensitivity represents the lowest analyte concentration that can be quantified with a coefficient of variation (CV) of less than 20%. Eleven serum pools covering the very low concentration range (<30 nmol/l) were tested in ten separate runs with a maximum of two runs per day.

### Serum and Plasma Sample Comparison

Serum and Li‐Heparin plasma sample combinations collected at one site (originating from the same patients) were analyzed in parallel as single determinations in a single run (*N* = 49 samples).

### Reagent Lot‐to‐Lot Reproducibility

Lot‐to‐lot reproducibility was evaluated at three sites using routine samples available. A total of 299 serum samples (approximately 100 samples per study site) were measured with three different reagent lots of the assay in single assay runs per reagent lot.

Lot‐to‐lot consistency was also evaluated at Amersfoort by monitoring the recovery of serum pools. For that, a low (approx. 20 nmol/l) and medium (approx. 60 nmol/l) serum pool was prepared, aliquoted, and stored at −80°C until the day of measurement.

### Method‐Correlation Studies

Correlation of the Elecsys assay with other methods for 25‐OHD quantitation was performed using residual routine patient samples available. Table 1 lists the method(s) of comparison and number of samples from each site. The LC‐MS/MS analysis at Heidelberg was performed on an Acquity HPLC system using an Acquity CSH‐C18 100 × 2.1 mm, 1.7 μm analytical column equipped with a Xevo TQ‐S mass spectrometer (Waters Corporation, Eschborn, Germany). Calibration was performed against Chromsystems standards (Munich, Germany) and NIST SRM 972 control material, using a modified version of a previously published LC‐MS/MS method 25. The Penzberg LC‐MS/MS method utilizes an HPLC system (Agilent 1100 series) with additional binary pump for online‐SPE (solid‐phase extraction) in conjunction with a triple quadrupole mass spectrometer with APCI source (atmospheric pressure chemical ionization; Thermo Scientific TSQ Quantum Ultra EMR). The calibration is performed against NIST standards as described above, using a modified version of a previously published LC‐MS/MS method 22. The HPLC system was calibrated with the Chromsystems Serum Calibration Standard D3/D3 nr 38033. To summarize, all LC‐MS/MS and HPLC methods used were calibrated against either NIST SRM 2972 or commercially available calibrators (Chromsystems, Munich, Germany), traceable to NIST SRM 2972 5, 21. Calibration of the immunoassays Liaison 25 OH Vitamin D total (DiaSorin, Inc. Stillwater MN) and IDS‐iSYS (Immunodiagnostic System, Boldon, UK) is traceable to UV spectrophotometry 26 and the assays were performed according to the manufacturers’ instructions.

### Statistical Methods

Between‐laboratory precision was analyzed by Variance Component Analysis (VCA) with fully nested design. Comparisons of serum and plasma samples were assessed using Pearson's coefficient of correlation (*r*) as determined by Passing–Bablok regression analysis 27. Reagent lot‐to‐lot comparisons and method comparisons were analyzed using Bland–Altman difference plots 28.

### Accuracy Testing Using the Vitamin D Reference Panel

The serum reference panel “Ref!25OHD” for 25‐OHD was obtained from Bioclin Oy—Labquality, Helsinki, and measured with the Elecsys assay on the cobas e 601 platform at Roche Diagnostics, Penzberg. The panel contains 20 serum samples from single donors certified by reference measurement procedures (RMPs) based on isotope dilution‐LC‐MS/MS (ID‐LC‐MS/MS, Prof. Linda Thienpont, University of Ghent).

## RESULTS

### Precision and Accuracy

Within‐run and intermediate precision of the Elecsys assay was assessed using two assay controls (PCV1 and PCV2) and three serum pools (SP1, SP2, SP3). For the low‐25‐OHD‐concentration range (SP1: 28.7 to 48.0 nmol/l; PCV1: 51. 9 nmol/l) the within‐run CVs ranged from 2.5 to 6.9%, for the medium concentrations (SP2: 81.9 to 90.6 nmol/l) the within‐run CVs ranged from 2.1 to 4.1%, and for the high concentrations (SP3: 102.7 to 131.8 nmol/l; PCV2: 114.0 nmol/l) the within‐run CVs ranged from 1.1 to 3.2% (Fig. 1, open circles).

Within‐laboratory (intermediate) precision resulted in CVs ranging from 3.6 to 9.3% (Fig. 1, closed circles). For the low‐concentration samples (SP1: 27.6 to 49.7 nmol/l; PCV1: 53 nmol/l) CVs were between 5.4 and 9.3%, for the medium concentrations (SP2: 82.1 to 90.6 nmol/l) between 4.4 and 6.7%, and for the high concentrations (SP3: 96.2 to 134.2 nmol/l; PCV2: 114.1 nmol/l) between 3.6 and 5.7%.

Results of the between‐laboratory precision testing, using aliquots prepared from three serum pools (SP4, SP5, and SP6), are presented in Table 2: the CV was 10.1% for the low‐25‐OHD‐serum pool (SP4: 35.6 nmol/l), and 5.8 and 7.5%, respectively, for the other two pools (SP5: 56.1 nmol/l and SP6: 94.8 nmol/l). The CVs for the between‐laboratory precision were within a similar range as those observed for the within‐laboratory (intermediate) precision (Fig. 1).

**Table 2:** Between‐Laboratory Precision and Recovery of 25‐OHD in Sample Pools

	Between‐laboratory precision				Percentage recovery based on between‐laboratory median value			
Sample	Mean (nmol/L)	Median (nmol/L)	SD (nmol/L)	CV (%)	Clayton	Wollongong	Munich	Amersfoort
**SP 4**	35.6	34.7	3.59	10.1	98.9	116.3	92.8	92.2
**SP 5**	56.1	54.8	3.23	5.8	99.6	109.7	100.6	96.3
**SP 6**	94.8	93.0	6.16	7.5	99.7	110.5	97.7	96.5

The recovery of 25‐OHD obtained for the three serum pools (SP4, SP5, and SP6) ranged from 92.2 to 116.3% of the median concentration of all four sites (Table 2). The between‐laboratory precision and recovery obtained in this study were compared with those of the independent survey Vitamin D External Quality Assessment Scheme (DEQAS, www.deqas.org). The DEQAS data for the first eight distributions including results reported with the Elecsys assay are summarized in Supplemental Figure 2. The number of participants using the Elecsys assay has increased from 26 laboratories in July 2011 to 121 laboratories (11% of the participating laboratories) in April 2013. The results obtained with the Elecsys assay as compared to the ALTM show a sample recovery of 86 to 131% and an overall mean of 102% for the eight quarterly time periods. The results obtained with the Elecsys assay as compared to the mean LC‐MS/MS values show a sample recovery of 72 to 119% and an overall mean of 95%. It is worth noting that the LC‐MS/MS methods used in this study are standardized to NIST material, whereas the standardization procedures for the LC‐MS/MS methods in the DEQAS are not known. Starting with October 2012 samples the total 25‐OHD (25‐OHD2 + 25‐OHD3) of DEQAS samples is determined in parallel by an NIST reference procedure and has been used as official target values from April 2013 onward. The recoveries of the Roche assay versus target values ranged between 91–99%, 80–108%, and 95–120% for October, January, and April distributions with mean recoveries of 96, 94, and 104%, respectively. The between‐laboratory precision of the Elecsys assay, as determined in the DEQAS multicenter evaluation survey, showed CVs of 13 to 22% at approximately 25 nmol/l, 6 to 12% at approximately 50 nmol/l, and 5 to 14% at approximately 75 nmol/l (Supplemental Figure 3).

### Functional Sensitivity

**Figure 2:**
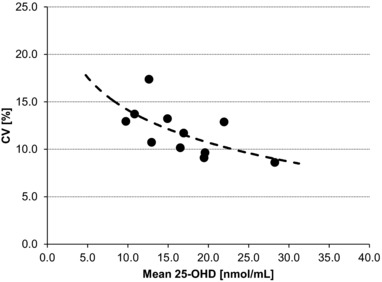
Functional sensitivity. Elecsys Vitamin D Total assay was used to analyze serum sample pools with low concentration of 25‐OHD in ten separate runs with a maximum of two runs per day, at Amersfoort. Median 25‐OHD concentrations were plotted against the corresponding CVs. To estimate the functional sensitivity, the data points were fitted using a logarithmic trend line.

Functional sensitivity of the assay was determined using serum pools from leftovers of routine samples with very low 25‐OHD concentrations (between 9.8 and 28.2 nmol/L). The CV of all measured samples was below 20% and the functional sensitivity for the Elecsys assay was determined as below 9.8 nmol/L corresponding to a CV of 12.9% (Fig. 2).

### Serum and Plasma Sample Comparison

Comparison of results from matched serum and Li‐Heparin plasma samples (*N* = 49) yielded a Passing–Bablok regression of *Y* = 0.959X − 0.344 (Pearson's correlation *r* = 0.988). The tested samples contained 25‐OHD in the concentration range of 15/ to 150 nmol/L.

### Reagent Lot‐to‐Lot Reproducibility

**Figure 3:**
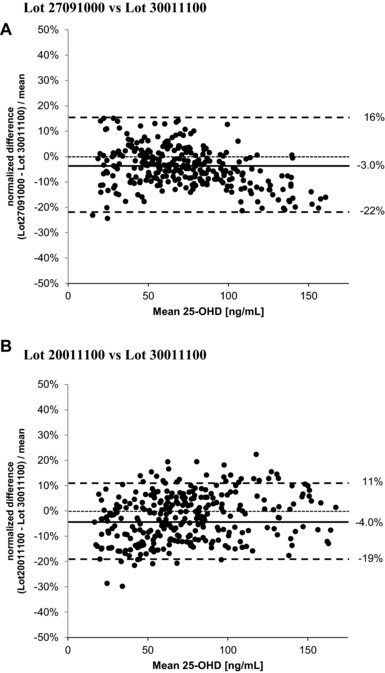
Lot‐to‐lot reproducibility (Bland–Altman analysis). Bland–Altman difference plots for lot‐to‐lot reproducibility evaluated at three sites, using routine samples available at each site. The center line (solid) represents the mean difference between the measurements, along with lines to mark the upper and lower limits of ±1.96SD (dotted lines), respectively.

Serum samples were analyzed with three lots of the Elecsys assay at three sites and the combined data were evaluated by Bland–Altman difference plots (Fig. 3). Comparing lot 27091000 *versus* lot 30011100 and lot 20011100 *versus* lot 30011100 yielded mean biases of −3% (±1.96 SD range from −22 to 16%) and −4% (±1.96 SD range from −19 to 11%), respectively.

**Figure 4:**
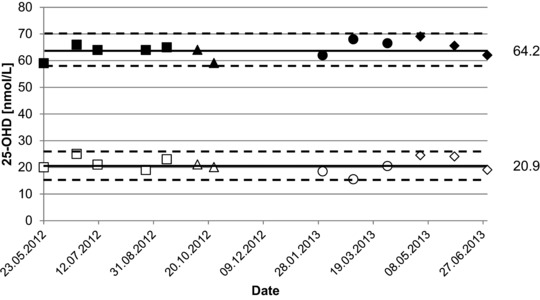
Long‐term recovery of serum pools. Lot‐to‐lot consistency was evaluated by monitoring the long‐term recovery of serum pools. A low (open symbols) and medium (filled symbols) was tested with different lots of Elecsys Vitamin D Total (□/▪ lot 163411, ▵/▴ lot 169889, ○/• lot 170827, ⋄/♦ lot 171102). The daily mean over two measuring cells was calculated.

Lot‐to‐lot consistency was also assessed at Amersfoort by monitoring the long‐term recovery of serum pools over 13 months covering four different lots of the Elecsys assay. For time intervals of approximately 4 weeks, the daily mean recovery over two measuring cells was calculated (see Fig. 4). The mean recoveries were 20.9 and 64.2 nmol/l with CVs of 12.5 and 4.6% for the low and medium serum pool, respectively.

### Comparison of Methods for 25‐OHD Quantitation

**Figure 5:**
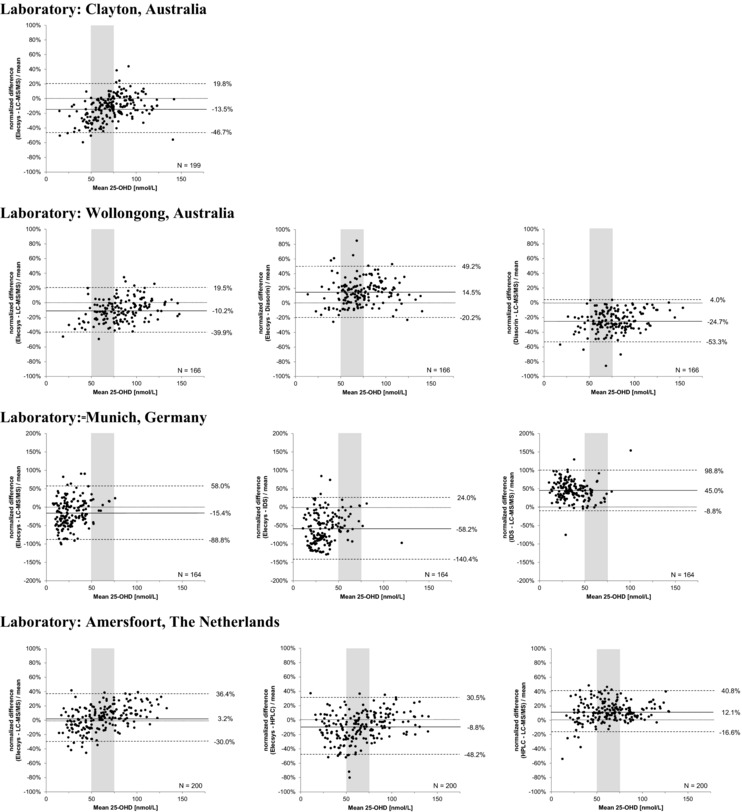
Comparison of 25‐OHD detection methods. Bland–Altman difference plots showing means of paired differences. The center line (solid) represents the mean difference between the measurements, along with lines to mark the upper and lower limits of ±1.96SD (dotted lines), respectively. The shaded area represents the clinically relevant concentration of 25‐OHD.

Performance of the Elecsys assay was compared with two chromatographic methods and two immunoassays as shown in Table 1. The Bland–Altman analysis (Fig. 5) showed biases ranging from −15.4 to 3.2% for the Elecsys assay *versus* LC‐MS/MS (±1.96 SD range from −97.1 to 69.5%). The majority of samples from the laboratory in Munich had concentrations of less than 50 nmol/l 25‐OHD. This may have contributed to the bias of −15.4% seen in comparison to LC‐MS/MS. Samples from Clayton and Wollongong analyzed with the same LC‐MS/MS method used to analyze the samples from Munich displayed biases of −13.5 and −10.2%, respectively. After the Elecsys testing in Amersfoort, samples were sent to Roche Diagnostics in Penzberg for LC‐MS/MS measurement. Comparison of these two methods yielded a relative mean bias of 3.2%. Bland–Altman analysis of the Elecsys assay versus HPLC showed a bias of −8.8% (±1.96 SD from −48.2 to 30.5%). Furthermore, comparison of the HPLC versus LC‐MS/MS showed a relative bias of 12.1% (±1.96 SD from −16.6 to 40.8%, laboratory in Amersfoort, Fig. 5).

Comparison of the Elecsys assay *versus* the DiaSorin Liaison immunoassay showed a positive relative bias of 14.5% (±1.96 SD from −20.2 to 49.2%, laboratory in Wollongong, Fig. 5). Comparison of the Roche Elecsys assay *versus* the IDS‐iSYS immunoassay showed a negative relative bias of −58.2% (±1.96 SD from −140.4 to 24.0%, laboratory in Munich, Fig. 5). Furthermore, comparison of 25‐OHD levels obtained with the DiaSorin Liaison *versus* LC‐MS/MS (laboratory in Wollongong, Fig. 5) showed a relative bias of −24.7% (±1.96 SD from −53.3 to 4.0%), indicating under recovery by the Liaison immunoassay. Comparison of the IDS‐iSYS *versus* LC‐MS/MS (laboratory in Munich, Fig. 5) showed a relative bias of 45.0% (±1.96 SD from −8.8 to 98.8%), by the IDS assay. For both immunoassays, the under or over recovery versus LC‐MS/MS is similar to the bias seen when the Elecsys assay was compared versus these assays.

### Accuracy Assessment for 25‐OHD2 and 25‐OHD3 Using the Vitamin D Reference Panel

**Figure 6:**
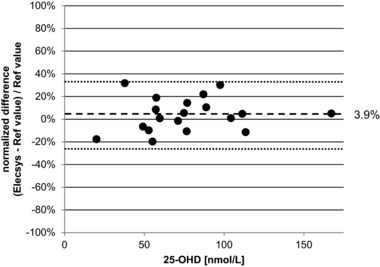
Assessment of the Vitamin D Reference Panel. The Vitamin D Reference Panel was analyzed by using a Bland–Altman difference plot. The dashed line represents the mean bias between Elecsys Vitamin D Total and the Reference values, the dotted lines indicate the upper and lower limits of ±1.96SD.

The Elecsys results for the reference panel were analyzed using Bland–Altman plots (Fig. 6). The normalized mean bias between the Elecsys assay and the reference values of the 20 samples was 3.9% (±1.96 SD from −25.3 to 33.2%). Five of the samples contained significant amounts of 25‐OHD2 (5.12 to 55.4 nmol/l equaling 9.6–48.8% of the total 25‐OHD). The normalized bias for these samples ranged from −11 to 5% with a mean of −3.4%, demonstrating close agreement with the ID‐LC‐MS/MS method (Thienpont, Ghent) also for 25‐OHD2.

## DISCUSSION

In the new ISO15189 guidelines as well as under European and American law, the responsibility of the manufacturer is to validate and to test a specified large set of method characteristics and the responsibility of the laboratory head is to test only a limited number of performance characteristics in their own lab, relying further on the work of the manufacturer in collaboration with a small group of peer laboratories. However, there is a rather large gap between the information about the performance characteristics the user should know and the ability of the manufacturer to condense this information in the kit insert. This study aims to close that gap, providing laboratories with more data to consider in assay selection.

One key element for establishing a reliable automated immunoassay for 25‐OHD is to ensure high consistency in the production of individual assay components. The use of polyclonal antibodies could lead to lot‐to‐lot variability and the risk of a different epitope recognition pattern upon immunization of different animals 7, 14, 15. Roche Diagnostics has substantial experience in developing competitive assays based on binding proteins. Hence, during the development of the Elecsys assay, it was decided to use a recombinant human VDBP rather than an antibody system. This provides a robust and stable system that mimics the “natural” binding mechanism of vitamin D. Another important and crucial step is an efficient and quantitative release of 25‐OHD from its binding protein. This is achieved by pre‐treating samples (serum or plasma) with protein denaturing reagents (dithiothreitol and sodium hydroxide) to irreversibly denature the endogenous VDBP in the sample. This also ensures that the endogenous binding protein does not interfere with the assay. A study investigating the influence of VDBP on the performance of 25‐OHD immunoassays confirmed that the Elecsys assay is not affected by special cohorts having elevated VDBP concentrations such as pregnant women 29.

Another key element for a reliable 25‐OHD assay is its standardization. The Elecsys assay is standardized against a modified version of the LC‐MS/MS method published by Vogeser et al. (22) with traceability to the reference material NIST SRM 2972, which consists of two ethanolic solutions of either 25‐OHD2 or 25‐OHD3 that are used to calibrate chromatographic methods 5, 21, 30. In addition, the performance of the in house LC‐MS/MS was monitored by using NIST SRM 972 (vitamin D in human serum), which is the first certified reference material for quality‐control use with officially assigned target values 5, 18, 19, 30. Hence, the calibration of the Elecsys assay is traceable to the NIST standards described above. This approach improves accuracy, reduces variability between the different methods, and contributes to global harmonization of 25‐OHD measurements 14, 15, 17, 18, 19.

The evaluation of the assay at four internationally located sites with substantial experience in vitamin D testing demonstrate that the Elecsys assay performed with a high level of within‐run precision over the clinically relevant range of 28.7 to 131.8 nmol/l, with CVs ranging from 1.1 to 6.9%. For within‐laboratory (intermediate) precision, the CVs ranged from 3.6 to 9.3% for all laboratories. Evaluation of between‐laboratory precision showed CVs of 5.8 to 10.1% for the 25‐OHD concentration range of 35.6 to 94.8 nmol/l. For a routine vitamin D assay, it has been calculated that the total imprecision (within‐laboratory precision 24) should be ≤10% and the data presented here show that the Elecsys assay fulfills this requirement (31). Recoveries of 25‐OHD, calculated as medians at individual sites *versus* median from all sites, ranged from 92.2 to 116.3%, indicating a good consistency and agreement for between‐laboratory measurements. The between‐laboratory precision as seen in the present MCE results is similar to the DEQAS results at the higher concentration, and a slightly better CV was observed for the pools with lower concentration (Table 2).

The low functional sensitivity (<9.8 nmol/l) demonstrates a high level of precision and is clinically relevant as it indicates that severely deficient patients can be identified. The Elecsys assay demonstrated a very good agreement between matched serum and plasma (*r* = 0.988), allowing flexibility in the sample type used for the measurement. Applying similar evaluation criteria, the assay also showed excellent reagent lot‐to‐lot reproducibility (*r* = 0.986 and *r* = 0.976). This was confirmed by monitoring the long‐term recovery of serum pools over a time period of 13 months. The recovery of serum samples showed little variation and was not affected by different assay lots used. This highlights the robustness of the assay and allows reliable monitoring of patients’ vitamin D status over time.

Correlation experiments of the Elecsys assay with other detection methods showed a normal distribution of data in the clinical decision range (shaded area from 50 to 75 nmol/l) in Figure 5. From the 2SD ranges for Amersfoort, Clayton, and Wollongong it can be derived that routine samples may show deviations of up to ±30% compared to LC‐MS/MS. This variation translates into correlation coefficients of 0.938, 0.874, and 0.893, respectively, which confirms the good overall comparability. However, the data from the site in Munich were primarily clustered at a concentration less than 50 nmol/l rather than over the entire measuring range. This may have contributed to the larger mean bias seen *versus* LC‐MS/MS for samples from this site (−25.4%) as compared to the mean biases seen from the other sites analyzed using the identical LC‐MS/MS instrument and method (−13.5 and −10.2%). A small positive bias versus LC‐MS/MS was observed with samples from Amersfoort (3.2%), in line with previously published results comparing the Elecsys assay against an LC‐MS/MS method used at Aarhus, Denmark (yielding a slope of 1.07 and intercept of 4.66 nmol/l, *r* = 0.89) 32. For Clayton and Amersfoort a slight concentration‐dependent bias trend is observed intersecting the *x*‐axis (0% bias) at approximately 50–75 nmol/l. This is generally seen if positive slopes and negative intercepts compensate each other concentration dependently. Taken overall, the data show negligible bias in the clinical decision range and good agreement between the assay and NIST‐standardized LC‐MS/MS measurements.

With its standardization, the Elecsys assay is in agreement with NIST traceable LC‐MS/MS or higher order reference methods as shown in the comparison studies using either routine samples or the official vitamin reference panel “Ref!25OHD.” For the latter, the difference plot of the Elecsys assay against the reference values showed a mean bias (3.9%) that was constant across the measuring range and is within the ±5% acceptance range for the bias as defined by Stöckl et al. 31. The good agreement of the Elecsys assay to the higher order reference method was also confirmed by the latest DEQAS results, since this scheme assigns values to their samples using an NIST Reference Measurement Procedure.

One limitation of our study design is the inability to address the question of 25‐OHD2 recovery using native samples from a patient population with sufficiently high 25‐OHD2 concentration. Samples spiked with 25‐OHD2 were not used in this evaluation, as some studies have shown that spiking with purified 25‐OHD2 can give underestimation of D2 recovery as compared with native plasma samples using competitive binding detection methods 33, 34, 35. As reported recently, the Elecsys assay shows underrecovery of 25‐OHD2 in native patient samples 17, 36. Conversely, it has been reported recently that the Elecsys assay has 101% cross‐reactivity with 25‐OHD2 in a study specifically designed to address the cross‐reactivity of commercial methods 37. Clinically, this discrepancy in the reports of 25‐OHD2 recovery is relevant for those countries that use vitamin D2 supplements. The vitamin D reference panel, which includes samples containing 25‐OHD2, is in our opinion the best referee concerning the cross‐reactivity for 25‐OHD2 of the Elecsys assay. The difference plot showed a mean bias of −3.4%, indicating a good recovery of samples containing 25‐OHD2 up to 50% of the total 25‐OHD.

An additional item to be addressed is the cross‐reactivity of the Elecsys assay toward 3‐epi‐25‐OH D3, which is present in 9–61% of the total 25‐OHD3 in the first year of life, and in 3–6% in adults 38. The biological activity of 3‐epi‐25‐OH D3 is still unclear. Recently it was demonstrated that the Elecsys assay shows no significant cross‐reactivity toward endogenous 3‐epi‐25‐OH D3, while approximately 50% cross‐reactivity toward exogenously added 3‐epi‐25‐OH D3 was observed 38. Hence the 3‐epimer does not interfere with the Elecsys assay in native samples.

Other recent publications based on the Elecsys assay have confirmed that the assay has good precision and accuracy, and shows close agreement to other well‐established methods for 25‐OHD analysis, making it very suitable for routine assessment of vitamin D status (32, 36, 39, 40). In conclusion, the Elecsys assay demonstrated low imprecision, high sensitivity, good lot‐to‐lot consistency, as well as good overall agreement with measurements obtained using LC‐MS/MS and HPLC methods. The question of 25‐OHD2 recovery was answered positively using the Thienpont reference serum panel. We have presented evidence that the Elecsys assay is suitable for routine automated measurement of 25‐OHD on multiple Roche instruments and will provide clinicians with a reliable assessment of vitamin D sufficiency.

## CONTRIBUTORSHIP

GFC collected data for the study, managed the study on site, and critically reviewed and approved the manuscript. HE designed the study, analyzed the data, and drafted and critically reviewed and approved the manuscript. GM collected data for the study, managed the study on site, critically reviewed and approved the manuscript. HJR analyzed samples by means of LC‐MS/MS, critically reviewed and approved the manuscript. CV contributed to study conception and design, statistical analysis and interpretation of the data, drafted and critically reviewed and approved the manuscript. JPMW analyzed samples and data, collected performance data, drafted and critically reviewed and approved the manuscript.

## Supporting information

Disclaimer: Supplementary materials have been peer‐reviewed but not copyedited.

**Figure S1**. Schematic representation of assay principle. Elecsys Vitamin D Total assay principle: Competitive protein binding assay
**Figure S2**. Summary of DEQAS data (July 2011 to April 2013). Vitamin D External Quality Assessment Scheme (DEQAS) results, reported with the Elecsys Vitamin D Total assay in comparison with the All‐Laboratory Trimmed Mean (ALTM) and the results reported by LC‐MS/MS. DEQAS analysis is based on unaltered samples only, therefore samples 405 and 414, spiked with 3epi‐25‐OHD and 24,25‐OH2D, were excluded from the analysis. Sample 430 with approximately 55% endogenous 25‐OHD2 was excluded from analysis as not all participants returned numerical results for both 25‐OHD2 and D3 for this sample.
**Figure S3**. Between‐laboratory precision, as determined by the Vitamin D External Quality Assessment Scheme (July 2011 to April 2013) for the results reported with the Elecsys Vitamin D Total assay.
