# Supplementary material for: Automated Competitive Protein‐Binding Assay for Total 25‐OH Vitamin D, Multicenter Evaluation and Practical Performance
Source: J Clin Lab Anal. 2014 Aug 17;29(6):451–61. doi: 10.1002/jcla.21793 (PMC6807057; doi:10.1002/jcla.21793)

**Automated competitive protein-binding assay for total 25-OH vitamin D, multi-center evaluation and practical performance**

**Authors:** JPM Wielders^1^, Graeme F Carter^2^, Heike Eberl^3^, Gary Morris^4^, Heinz Jürgen Roth^5^, Christian Vogl^3^

**Short title:** Multi-center evaluation of total 25-OHD assay

**SUPPLEMENTARY MATERIAL** **FOR REVIEW**

Supplemental Figure 1. Schematic representation of assay principle.

Supplemental Figure 2. Summary of DEQAS data (July 2011 to April 2013).


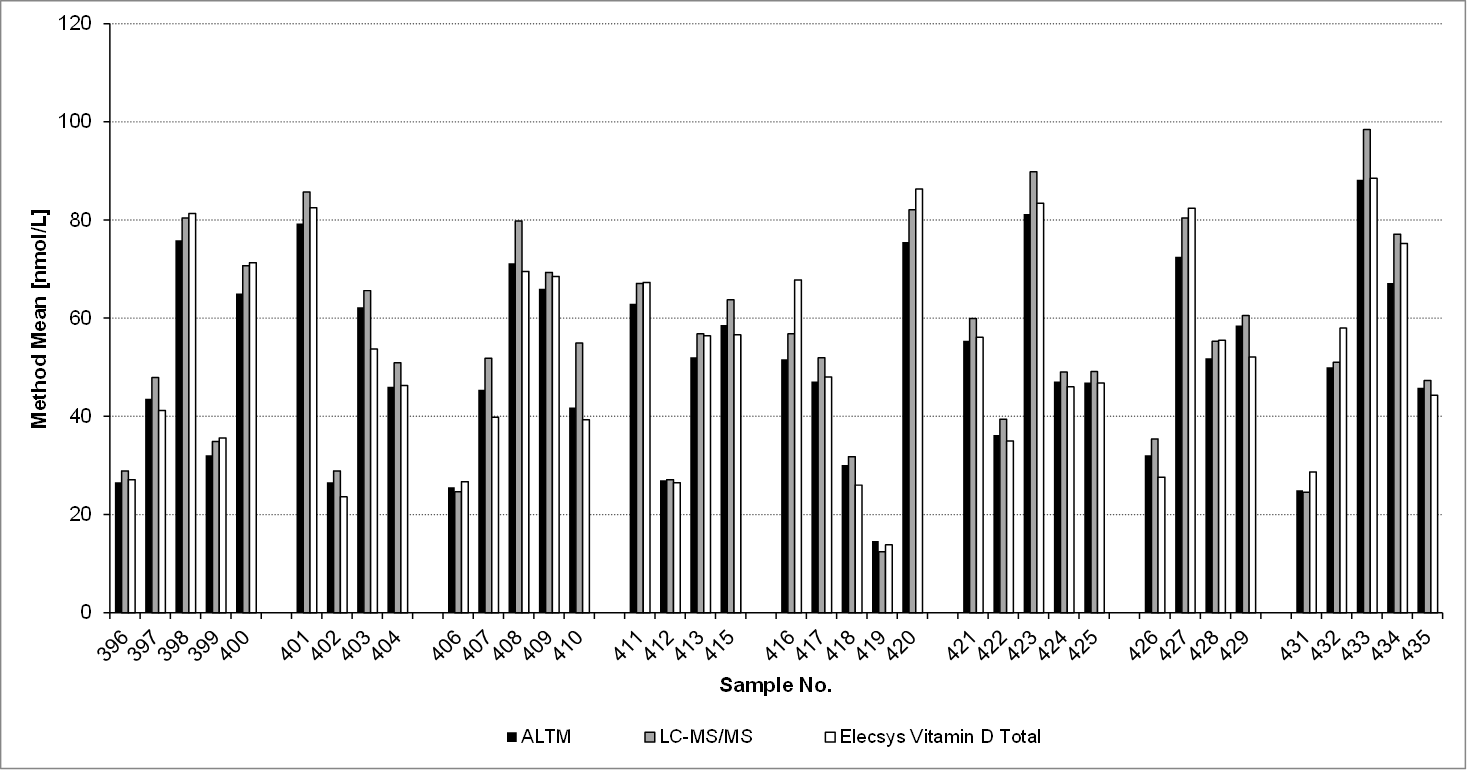


Supplemental Figure 3. Between-laboratory precision, as determined by the Vitamin D External Quality Assessment Scheme (July 2011 to April 2013) for the results reported with the Elecsys Vitamin D Total assay.


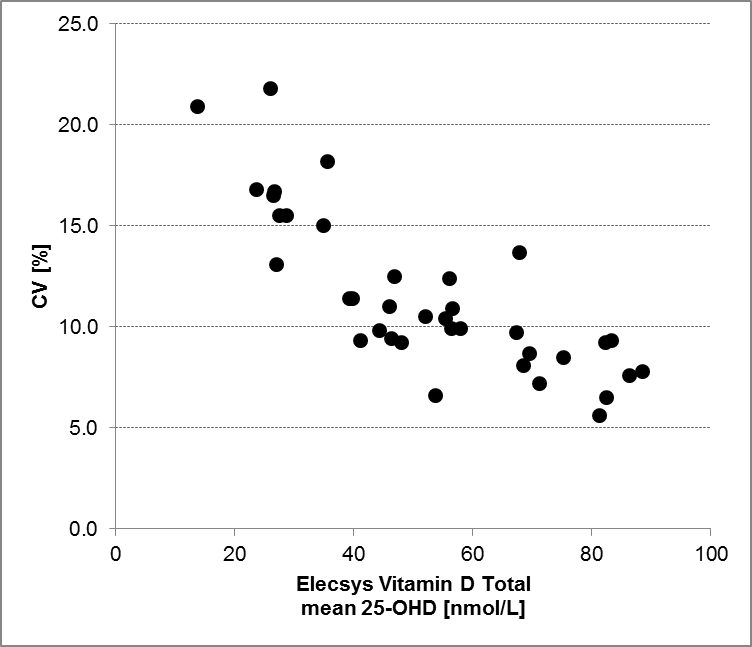

Supplement: Supplementary file 1 — Figure S1. Schematic representation of assay principle. Elecsys Vitamin D Total assay principle: Competitive protein binding assay Figure S2. Summary of DEQAS data (July 2011 to April 2013). Vitamin D External Quality Assessment Scheme (DEQAS) results, reported with the Elecsys Vitamin D Total assay in comparison with the All‐Laboratory Trimmed Mean (ALTM) and the results reported by LC‐MS/MS. DEQAS analysis is based on unaltered samples only, therefore samples 405 and 414, spiked with 3epi‐25‐OHD and 24,25‐OH2D, were excluded from the analysis. Sample 430 with approximately 55% endogenous 25‐OHD2 was excluded from analysis as not all participants returned numerical results for both 25‐OHD2 and D3 for this sample. Figure S3. Between‐laboratory precision, as determined by the Vitamin D External Quality Assessment Scheme (July 2011 to April 2013) for the results reported with the Elecsys Vitamin D Total assay. [file JCLA-29-451-s001.doc]
